# Emergency-related inpatient admissions in child and adolescent psychiatry: comparison of clinical characteristics of involuntary and voluntary admissions from a survey in Bavaria, Germany

**DOI:** 10.1007/s00787-023-02154-3

**Published:** 2023-02-16

**Authors:** Stephanie Kandsperger, Angelika Ecker, Daniel Schleicher, Michael Wirth, Romuald Brunner, Irina Jarvers

**Affiliations:** Clinic of Child and Adolescent Psychiatry, Psychosomatics and Psychotherapy, University Hospital of Regensburg, Regensburg, Germany

**Keywords:** Inpatient admissions, Emergency, Adolescents, Mental health, Involuntary presentations, Accompanying persons

## Abstract

Emergency inpatient admissions of children and adolescents are more difficult if the patient is admitted involuntarily and/or the caregivers or custodians of institutional care are absent. The present study aimed to clinically characterize involuntary versus voluntary admissions by examining the reasons for presentation and associated factors. We retrospectively analyzed patients who presented to the emergency department of a hospital for child and adolescent psychiatry in Bavaria, Germany, and were admitted as inpatients for crisis intervention in the 4th quarter of 2014–2018. Reasons for presentation, clinical and sociodemographic characteristics, and type of admission (voluntary versus involuntary) were analyzed for 431 emergency inpatient admissions. A total of 106 (24.6%) patients were involuntarily admitted. In a binominal logistic regression, presentation due to alcohol consumption, deviant social behavior, and psychosocial burden was positively associated, whereas difficulties at school and depression were negatively associated, with the likelihood of involuntary admission. 58.5% of the 123 unaccompanied patients were admitted involuntarily. Reasons for the presentation of unaccompanied and voluntary inpatient admissions were suicidal thoughts, psychosocial burden, and externalized aggression. A substantial number of child and adolescent psychiatric admissions represent emergency admissions. Involuntarily admitted patients and unaccompanied children/adolescents represent a non-negligible proportion of clinical routine and the clinical and legal background factors need to be further clarified in future studies. This study is registered in the German Clinical Trials Register (24 September 2019, DRKS00017689).

## Introduction

Children and adolescents presenting to child and adolescent psychiatry on an emergency basis show a broad range of concerns, ranging from minor or moderate psychiatric symptoms to conditions that are potentially life-threatening [1, 2]. If left untreated, such emergencies may result in damage either to the persons themselves or to others around them [3]. In a retrospective data collection in a hospital for child and adolescent psychiatry in Germany, it was found that in 66% of crisis patients a mental health problem was already present before the crisis [4]. Furthermore, in half of the crisis patients examined, symptoms leading to crisis intervention had only been present for a few days, whereas the other half stated that the crisis condition had been present for weeks or even chronically [4]. In recent years, emergency presentation of children and adolescents for mental health reasons has increased, as papers from several nations report [2, 5, 6]. A cohort study of inpatient treatment (regular and emergency admission) at child and adolescent psychiatric hospitals in Germany detected an increase in inpatient treatment [7]. A retrospective data analysis between 1996 and 2014 from a child and adolescent psychiatric hospital in Germany showed a 405% increase in emergency admissions as well as a higher amount of adverse psychosocial background factors and more challenging disease situations in later years [8]. In another study from Germany an increase of 219% of emergency admissions to a child and adolescent psychiatric hospital that occurred between 3 p.m. and 8 a.m., has been reported during the period between 2005 and 2015: from 116 (21.2% of all admissions) to 370 (41.0% of all admissions) admissions [9]. Although hospitalization can be a major disruption for children and adolescents, it is sometimes essential to treat young people experiencing a psychiatric crisis in an inpatient setting [10].

Thus, every emergency child and adolescent psychiatric presentation needs immediate assessment regardless of whether an inpatient admission for crisis intervention is indicated. A previous study from Germany reported that 47.1% of patients presenting as emergencies were admitted to an inpatient unit [6]. Caregivers being present at the emergency department increased the likelihood of treatment alternatives for immediate admission if they presented outside of regular working hours [11]. This points to the fact that accompanying factors may also have a significant impact on whether a presented patient is admitted directly as an inpatient [11]. In the absence of parents, there is often missing information for example about familial resources and the current situation of the patient [11]. Involving parents or other caregivers, which is typically required for children and adolescents, usually means that assessment of the severity of the acute crisis and potential conflict resolution options are made considering a young person's environment [12]. Further age-related challenging factors arise from the fact that emergencies are usually defined as such by an individual different from the child or adolescent involved, and that a variety of potential caregivers may be involved [12].

Normally, children and adolescents are admitted and treated voluntarily with patients and their legal guardians agreeing to the admission and treatment [7]. In Germany, the harm of not being treated justifies the involvement of the family court for a placement order, and an application to the court by their legal guardians and a medical recommendation by a doctor are required to admit and treat legally. In Germany, these decisions are made based on Sect. 1631b of the German Civil Code (BGB) [13]. According to this law, placement of the child involving restriction of civil liberties requires approval by the family court. Furthermore, placement is only permissible as long as it is necessary for the child's welfare, particularly to avoid significant risk to the child or others, and the risk cannot be countered in any other way, including other public assistance, such as institutional placement [13, 14]. Under this legal basis, patients are usually admitted promptly as inpatients, but in a planned manner, as the court order is often obtained prior to the admission. The principle is that a hospital is not obligated to admit the patient if the physician on duty does not determine a need for immediate admission.

According to the mental health laws (Psych-KG) of the individual federal states of Germany, there is also the possibility of restraint in an emergency in the inpatient setting [14]. According to the Bavarian detention law, the following applies: “Any person who is mentally ill or mentally disturbed as a result of mental weakness or addiction and thereby poses a considerable danger to public safety or order may be placed against or without his will in a psychiatric hospital or elsewhere in a suitable manner (BayUnterbrG)” [15]. In particular, placement is also permissible if a person endangers his or her life or, to a considerable extent, his or her health, but placement may only be ordered if the danger cannot be prevented by less drastic means [15]. In urgent cases, police may admit the person immediately to a psychiatric institution [15, 16]. If an examination of the patient at the hospital shows that necessary conditions are not met, the affected person must not be detained against their will [15]. Otherwise, the person must be presented to the family court without delay, no later than the day after the beginning of the detention [15]. This Bavarian detention law (BayUnterbrG) was valid until 31 December 2018 (i.e., during our data collection period). The new law, BayPsychKHG, came into use on 1 January 2019 for immediate temporary detentions [17]. There is a legal hierarchy, prioritizing Civil Court Orders (BGB) over BayPsychKHG for children and adolescents [17]. The ethical and clinical aspects of involuntary treatment are controversial [18] and outside the scope of this article.

Many studies [2, 19, 20] have reported emergency admissions and associated factors independent of voluntary/involuntary inpatient treatment, but few studies have compared voluntary and involuntary inpatient admissions of children and adolescents after an emergency presentation [10]. For example, in a retrospective survey of all hospital admissions of adolescents in psychiatric hospitals (1996–2003) in Finland, involuntary inpatient treatment increased 1.6-fold [21]. In a large cohort study of voluntary and involuntary treatment (regular or emergency admission) at child and adolescent psychiatric hospitals in Germany, the proportion of involuntary admissions decreased from 32.4% in 2004 to 25.7% in 2009 [7]. Strong factors associated with involuntary admission in Germany were the presence of intellectual disability, adolescent age, substance abuse, psychotic disorders, and hospitalization outside regular working hours [7]. Compared to voluntary admission, involuntary admission of 6- to 18-year-olds after emergency presentation in the Netherlands was related to more severe psychiatric problems, higher risk of suicide, and earlier emergency involuntary admission [10]. In a Finnish study, involuntary adolescent admissions to a child and adolescent psychiatric hospital were related to patient symptom severity (i.e., psychotic symptoms, violent behaviors, and suicidal ideation) but not to aspects of the adolescents' life circumstances (i.e., family adversities, previous treatment, or sociodemographic factors) [22]. Retrospective data on adolescents in a psychiatric crisis in Italy showed that involuntary treatments were initiated, especially in the case of conduct disorders and comorbid diagnoses [23].

The current study retrospectively investigated the sociodemographic and clinical characteristics of emergency presentation of children and adolescents who were directly admitted to a psychiatric inpatient unit. This study covered a consecutive period of 5 years, utilizing data from the 4th quarter of each year. Reasons for emergency presentation and subsequent admission were examined. We also compared patients admitted voluntarily on an emergency basis with patients who were involuntarily placed (BGB or BayUnterbrG). Therefore, we examined a wide range of characteristics that may be associated with an involuntary crisis intervention admission. We assumed that mainly patients with externalizing behaviors would be involuntarily admitted to inpatient units. Furthermore, physicians on duty advised that parents or guardians and/or caregivers from an (outpatient) youth welfare institution or responsible staff member of the youth welfare office should accompany the child for emergency presentation. To the best of our knowledge, there has been little research on this patient group, namely children and adolescents who present as emergencies and are admitted as inpatients involuntarily and/or unaccompanied by parents/caregivers/youth care workers.

## Materials and methods

We retrospectively collected data on all children and adolescents who consecutively presented face-to-face on an emergency basis to a large child and adolescent psychiatric hospital in Germany (Bavaria) and were admitted as inpatients in an acute crisis or sent for emergency inpatient admission by colleagues from branch offices, private practices, or other medical services. These referrals are regularly checked for the necessity of inpatient admission. The hospital is a typical child and adolescent psychiatric hospital of maximum care with three inpatient units with 28 beds (40 beds starting in October 2017). During regular opening hours, the hospital is responsible for the emergency care of children and adolescents from the city of Regensburg and several suburban regions. Outside regular treatment hours, the clinic provides emergency services to the entire Upper Palatinate region. The Upper Palatinate is a government district of Bavaria with 1,112,267 inhabitants [24].

Emergency service at the hospital runs 24/7 and was recorded retrospectively in the years 2014 to 2018 (4th quarter each, from October 1 to December 31). The 4th quarter was selected because it shows a representative presentation rate and ensures comparability. The study aimed to investigate the course of admissions over time while keeping the documentation and coding of cases to a manageable amount. The basis for the selection of patients in the data collection was provided by the daily documentation sheets of the physicians on duty, which contains basic data of the emergency patients (date, name of patients, emergency presentation, inpatient admission, and admitting unit). Based on this, we were able to utilize the scanned handwritten notes of physicians on duty, existing outpatient and inpatient process documentation, and existing doctors’ letters from outpatient and inpatient treatment in the electronic hospital documentation system. This allowed a coherent evaluation of the content of the emergency presentations and admissions. Sociodemographic and clinical characteristics of inpatient admissions were collected and are presented in Tables 1 and 2. Psychiatric diagnoses were assigned according to chapter V (F) of the International Classification of Diseases (ICD-10) [25]. In addition, we recorded reasons that initially led to emergency presentation and finally inpatient admission, as well as the legal context of the treatment (voluntarily, BGB, BayUnterbrG). For the variable “psychosocial burden” as a reason for presentation, we have taken axis V of the multiaxial classification scheme for mental disorders of childhood and adolescence according to ICD-10 [26]. In addition, we recorded whether patients came accompanied (with parents, guardians, close relatives, or institutional caregivers) or unaccompanied (“alone”). An escort by police officers or ambulance/emergency doctor was not subsumed here.

**Table 1:** Demographic description of the sample of emergency inpatient admissions in the 4th quarter of the years of observation

Variable	Years of observation					Total
	2014	2015	2016	2017	2018	
Number of cases	58	89	69	114	101	431
Age, years						
M	15.34	15.43	15.50	15.26	15.33	15.36
SD	1.79	2.28	1.79	1.80	2.17	1.99
Range	11.31–18.01	6.29–17.97	9.30–17.96	10.22–18.29	4.28–17.97	4.28–18.29
Female patients	37 (63.8)	46 (51.7)	35 (50.7)	69 (60.5)	68 (67.3)	255 (59.2)
Living area						
Rural community	15 (25.9)	23 (25.8)	17 (24.6)	25 (21.9)	21 (20.8)	101 (23.4)
Small city	22 (37.9)	25 (28.1)	26 (37.7)	42 (36.8)	35 (34.7)	150 (34.8)
Medium city	8 (13.8)	21 (23.6)	10 (14.5)	18 (15.8)	29 (28.7)	86 (20.0)
Big city	13 (22.4)	20 (22.5)	16 (23.2)	29 (25.4)	16 (15.8)	94 (21.8)
Refugees	4 (6.9)	17 (19.1)	8 (11.6)	4 (3.5)	3 (3.0)	36 (8.4)
Involuntary emergency admissions (BayUnterbrG or BGB)	9 (15.5)	26 (29.2)	16 (23.2)	21 (18.4)	34 (33.7)	106 (24.6)
BayUnterbrG	6 (66.7)	25 (96.2)	16 (100.0)	15 (71.4)	32 (94.1)	94
BGB	3 (33.3)	1 (3.8)	0 (0.0)	2 (9.5)*	2 (5.9)	8*
Patients known to clinic	33 (56.9)	60 (67.4)	49 (70.0)	75 (65.8)	73 (72.3)	289 (67.1)
Repeated presentation in the same quarter	16 (27.5)	23 (25.8)	15 (21.4)	22 (19.3)	20 (19.8)	96 (22.3)

Values are given as *n* (%) unless otherwise noted. Living area classification was based on the population: up to under < 5000 inhabitants = Rural Community; 5000 to < 20,000 inhabitants = Small City; 20,000 to < 100,000 inhabitants = Medium City; > 100,000 inhabitants = Big City

*In *n* = 4 cases from 2017 it could not be determined whether BayUnterbrG or BGB applied

**Table 2:** Clinical description of the sample in the 4th quarter of the years of observation

Variables	Years of observation					Total
	2014	2015	2016	2017	2018	
Reasons for presentation						
School problems	10 (17.2)	10 (11.2)	12 (17.4)	16 (14.0)	25 (24.8)	73 (16.9)
Alcohol consumption	1 (1.7)	5 (5.6)	12 (17.4)	13 (11.4)	14 (13.9)	45 (10.4)
THC consumption	4 (6.9)	5 (5.6)	3 (4.3)	16 (14.0)	11 (10.9)	39 (9.0)
Other substances	5 (8.6)	7 (7.8)	4 (5.7)	11 (9.6)	16 (15.8)	43 (10.0)
Deviant social behavior	14 (24.1)	24 (27.0)	22 (31.9)	35 (30.7)	28 (27.7)	123 (28.5)
Externalized aggression	9 (15.5)	26 ( 29.2)	19 (27.5)	26 (22.8)	25 (24.8)	105 (24.4)
ADHD symptomatology	0 (0.0)	0 (0.0)	4 (5.8)	3 (2.6)	1 (1.0)	8 (1.9)
Anxious symptomatology	6 (10.3)	4 (4.5)	3 (4.3)	8 (7.0)	12 (11.9)	33 (7.7)
Depressive symptomatology	15 (25.9)	18 (20.2)	20 (29.0)	50 (43.9)	27 (26.7)	130 (30.2)
Eating behavior problems	2 (3.4)	3 (3.4)	2 (2.9)	5 (4.4)	4 (4.0)	16 (3.7)
Psychotic symptomatology	4 (6.9)	5 (5.6)	7 (10.1)	8 (7.0)	3 (3.0)	27 (6.3)
Obsessive–compulsive symptomatology	0 (0.0)	0 (0.0)	0 (0.0)	1 (0.9)	1 (1.0)	2 (0.5)
Psychosocial burden	25 (43.1)	39 ( 43.8)	35 (50.7)	45 (39.5)	47 (46.5)	191 (44.3)
Non-suicidal self-injury	16 (27.6)	25 (28.1)	23 (33.3)	33 (28.9)	30 (29.7)	127 (29.5)
Suicidal thoughts	45 (77.6)	54 (60.7)	45 (65.2)	79 (69.3)	68 (67.3)	291 (67.5)
Suicide attempts	8 (13.8)	10 (11.2)	5 (7.2)	3 (2.6)	6 (5.9)	32 (7.4)
Somatic symptomatology	5 (8.6)	2 ( 2.2)	2 (2.9)	7 (6.1)	5 (5.5)	21 (4.9)
Sexual abuse	2 (3.4)	0 (0.0)	3 (4.3)	1 (0.9)	2 (2.0)	8 (1.9)
Child maltreatment	0 (0.0)	1 (1.1)	1 (1.4)	0 (0.0)	1 (1.0)	3 (0.7)
Diagnosis groups						
F1	4 (6.9)	10 (11.2)	14 (20.3)	32 (28.1)	23 (22.8)	83 (19.3)
F2	2 (3.4)	0 (0.0)	5 (7.4)	2 (1.8)	1 (1.0)	10 (2.3)
F3	23 (39.7)	49 ( 55.1)	50 ( 73.5)	59 (51.8)	58 (57.4)	239 (55.6)
F4	29 (50.0)	34 (38.2)	19 (27.9)	41 (36.0)	36 ( 35.6)	159 (37.0)
F5	4 (6.9)	3 (3.4)	4 (5.9)	6 (5.3)	2 (2.0)	19 (4.4)
F6	5 (8.6)	13 (14.6)	5 (7.4)	10 (8.8)	6 (5.9)	39 (9.1)
F7	0 (0.0)	0 (0.0)	0 (0.0)	0 (0.0)	0 (0.0)	0 (0.0)
F8	0 (0.0)	2 (2.2)	0 (0.0)	2 (1.8)	0 (0.0)	4 (0.9)
F9	20 (34.5)	35 (39.3)	31 (44.9)	60 (52.6)	44 (43.6)	190 (44.1)
Multiple diagnoses	27 (46.6)	60 (67.4)	47 (68.1)	78 (68.4)	70 (69.3)	282 (65.4)

Values are given as *n* (%). Reasons for presentation and F-diagnoses do not add up to 100% due to multiple reasons for presentation/diagnosis being possible

### Statistical analysis

In a first step, the change in emergency inpatient admissions was examined via a Poisson regression with number of cases per month as the dependent variable and time (in months), increase in available beds (“Beds”), and number of refugees (“Ref”) as independent variables. Poisson regression was chosen because the estimated dispersion coefficient in a negative binominal regression was close to zero. Time was not coded successively, but with breaks for missing months in order to ensure accurate calculation of the slope for the regression. Bed increase was included because the hospital was extended by 12 beds in October 2017 and more inpatient admissions were possible (coded 0/1 per month). In a second step, emergency cases that were admitted involuntarily were examined for changes in frequency over time. A Poisson regression was computed with the number of involuntarily admitted cases per month as the dependent variable and time, number of total cases per month, and Ref as independent variables. Ref was added as a control variable because a higher proportion was observed around the year 2015 due to the Syrian war. For all regressions, model assumptions were checked and exponentiated parameter estimates were reported (incidence rate ratio [IRR]).

Group comparisons between inpatients who were admitted voluntarily vs. involuntarily were computed. For metric and normally distributed variables, *t* tests were computed. For non-normally distributed dependent variables, Mann–Whitney *U* tests were used. Non-metric variables were tested by *χ*^2^ tests of independence, which were also used to determine whether certain reasons for presentation were more likely to result in involuntary inpatient admission. Finally, to determine variables significantly associated with involuntary admission, a binominal logistic regression was computed with variables that showed significant differences between the groups as independent variables. For tests examining group differences, the false discovery rate (FDR) was used to correct for multiple comparisons [27]. The FDR controls the expected proportion of false positives. Advantages compared to other corrections are a more specific application and, thereby, higher power. Reported *p* values already correspond to the correction. Statistical analyses were conducted using SPSS 28 (IBM SPSS Statistics for Windows, Version 28.0. Armonk, NY: IBM Corp.), and the statistical significance set to *α* = 0.05.

## Results

### Sample

Overall, 431 emergency presentations were admitted to the inpatient unit in the 4th quarter of the years 2014 to 2018. The sociodemographic and clinical characteristics of inpatient admissions are presented in Tables 1 and 2.

### Trend of inpatient admissions over time

A Poisson regression was conducted to identify frequency changes in inpatient admissions between 2014 and 2018. The number of cases each month was chosen as the dependent variable and Beds, time (in months), and Ref as independent variables. The model fits significantly better than the null model (*χ*^*2*^(3) = 24.55, *p* < 0.001). Of the three independent variables, only Bed and Ref were significantly positively associated. Bed increase was associated with a 79.40% increase in cases and, for each additional presentation of a refugee, a 7.60% increase in cases was observed (Table 3).

**Table 3:** Poisson regression models assessing number of inpatient and involuntary admissions

Dependent variable	Predictor	IRR	*p*	95% CI
Number of inpatient admissions	Time	1.00	0.987	0.99–1.01
	Bed increase	1.79	0.009	1.16–2.79
	Refugees	1.08	0.006	1.02–1.13
Number of involuntary inpatient admissions	Time	1.02	0.008	1.01–1.04
	Number of inpatient admissions	1.01	0.458	0.98–1.04
	Refugees	1.13	0.022	1.02–1.25

Bed increase refers to an increase in inpatient beds starting in October 2017

*IRR* incidence rate ratio, *CI* confidence interval

**Fig. 1:**
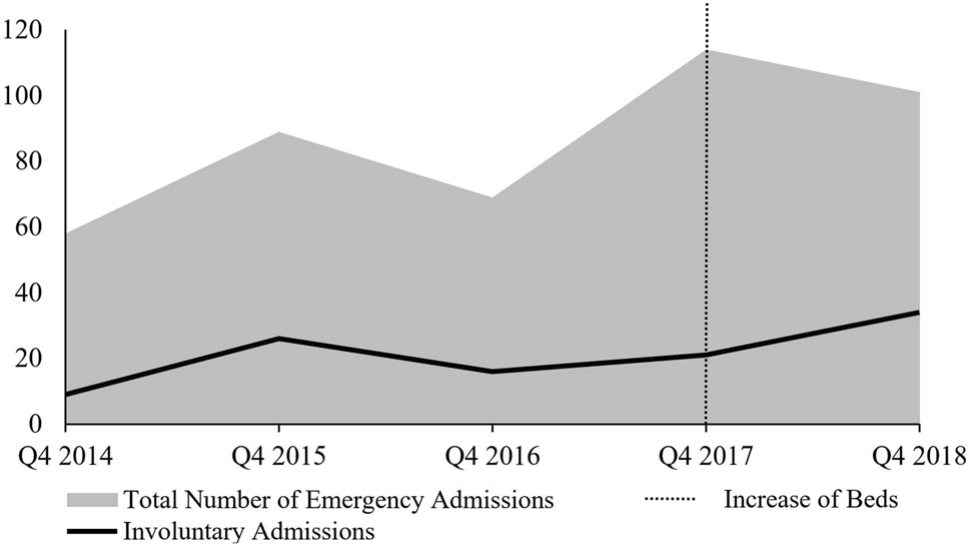
Time course of emergency inpatient admissions and proportionate involuntary admissions. Q4 = 4th quarter of each year. 2017 saw an increase of 12 beds in the inpatient department

To examine frequency change in involuntary admissions over time, a second Poisson regression model was computed with number of involuntary inpatient admissions each month as the dependent variable and time (in months), overall number of admissions each month, and Ref as independent variables. The model fits significantly better than the null model (*χ*^*2*^(3) = 16.71, *p* < 0.001). Among the independent variables, only time and Ref were significantly positively associated. A 1-unit increase in time (in months) resulted in a 2.00% increase in involuntary admissions and, for each additional presentation of a refugee, a 13.00% increase in involuntary admissions was observed (Table 3). Among refugees (*N* = 36), 21 (58.3%) came with placement orders, 2 are according to BGB and 19 according to BayUnterbrG. Figure 1 depicts emergency inpatient admissions and involuntary admissions between 2014 and 2018, aggregated over the examined quarters.

### Group differences between voluntary and involuntary inpatient admissions

Mann–Whitney *U* and *χ*^2^ tests were used to examine group differences in age, sex, length of stay (in days), Ref, and involuntary inpatient admissions (Table 4). Overall, there was no difference in age, but involuntary inpatient admissions were more likely to be male, have shorter lengths of stay, and a higher likelihood of being a refugee.

**Table 4:** Group comparisons between voluntary and involuntary inpatient admissions

Variable	Voluntary admissions	Involuntary admissions	Test statistic
Age	15.48 (2.08)	15.85 (1.79)	*U* = 15,704.00
			*z* = − 1.37
			*p* = 0.172^a^
Sex	Male = 111 (34.15%)	Male = 41 (38.68%)	*χ*^2^ = 24.42
	Female = 214 (65.85%)	Female = 65 (61.32%)	*p* < 0.001^b^
Length of stay (days)	13.23 (32.30)	6.64 (10.02)	*U* = 13,235.50
			*z* = − 3.61
			*p* < 0.001^a^
Refugees	15 (4.62%)	21 (19.81%)	*χ*^2^ = 24.11
			*p* < 0.001^b^

*p* values have been FDR-corrected

^a^Mann–Whitney *U* test

^b^*χ*^2^ test of independence

**Fig. 2:**
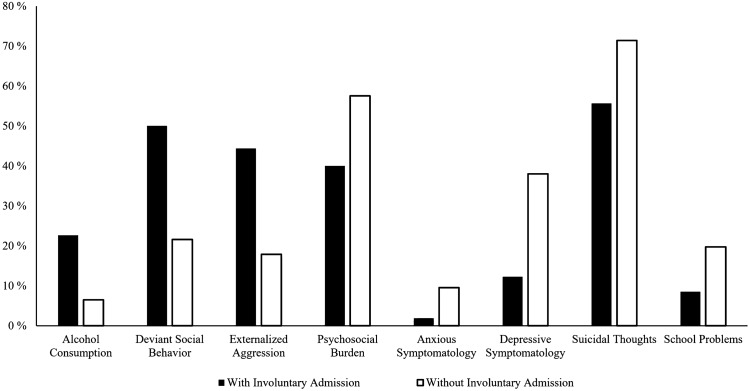
Comparison of the reasons for presentation with and without involuntary admission. Percentages of patients with the reasons for presentation with significant group differences (*p* < 0.01) between inpatients with and without involuntary admission are shown. No significant differences were found in the presentation reasons “THC Consumption”, “Other Substances”, “Non-Suicidal Self-Injury”, “Suicide Attempts”, “Somatic Symptomatology”, and “Psychotic Symptomatology”. Multiple reasons for presentation could be given

Differences in the reasons for presentation were examined by *χ*^2^ tests comparing whether a reason was more frequent among involuntary compared to voluntary admissions. Only reasons that occurred in > 5.0% of the sample were considered. Expected cell counts were never < 5, with the smallest expected cell count being 5.58. The reasons school problems, depressive symptomatology, and suicidal thoughts were associated with fewer involuntary admissions, whereas alcohol consumption, deviant social behavior, externalized aggression, and psychosocial burden were associated with more involuntary admissions. Figure 2 shows a frequency distribution of reasons for presentation, with significant differences between the two groups. There was no significant difference between involuntary and voluntary inpatient admissions due to THC consumption (*χ*^2^ = 0.30, *p* = 0.563), other substance consumption (*χ*^2^ = 2.14, *p* = 0.192), non-suicidal self-injury (*χ*^2^ = 0.04, *p* = 0.851), suicide attempts (*χ*^2^ = 0.64, *p* = 0.425), somatic symptomatology (*χ*^2^ = 3.62, *p* = 0.057), and psychotic symptomatology (*χ*^2^ = 3.65, *p* = 0.056).

### Reported reasons for presentation significantly associated with involuntary admission

Significant reasons for presentation were simultaneously entered into a binominal-logistic regression (Table 5) to assess their effect on the likelihood of involuntary inpatient admission. The logistic regression model was significant (*χ*^2^(11) = 106.74, *p* < 0.001). The model explained 32.6% (Nagelkerke R^2^) of the variance in involuntary admissions and correctly classified 79.1% of cases. The model had a specificity of 82.37%, a sensitivity of 61.76%, a positive predictive value of 39.60%, and a negative predictive value of 92.00%. Being a refugee or presenting due to problems in school, alcohol consumption, deviant social behavior, depressive symptomatology, and psychosocial burden was significantly (positively or negatively) associated with involuntary admission. Refugees were 274.10% more likely to be admitted involuntarily. Patients presenting due to problems in school were 65.30% less likely, due to alcohol consumption 406.70% more likely, due to deviant social behavior 238.70% more likely, with psychosocial burden 92.00% more likely, and due to depressive symptomatology 61.60% less likely to be admitted involuntarily.

**Table 5:** Multivariate binominal logistic regression assessing the likelihood of involuntary inpatient admission

Dependent variable	Predictor	Exp* (B)*	95% CI	*p*
Likelihood of involuntary inpatient admission	Sex	1.61	0.92–2.81	0.096
	Refugees	2.74	1.15–6.55	0.023
	School problems	0.35	0.14–0.86	0.022
	Alcohol consumption	4.07	1.97–8.40	< 0.001
	Anxious symptomatology	0.33	0.07–1.52	0.153
	Depressive symptomatology	0.38	0.19–0.78	0.008
	Deviant social behavior	2.39	1.37–4.17	0.002
	Externalized aggression	1.74	0.96–3.17	0.070
	Psychosocial burden	1.92	1.14–3.24	0.015
	Suicidal thoughts	0.84	0.47–1.50	0.549
	Length of stay (days)	0.99	0.97–1.01	0.213

*CI*  confidence interval. All predictor variables were added simultaneously

### Characterization of unaccompanied and voluntary inpatient admissions

Finally, unaccompanied inpatient admissions were examined in detail. A total of 123 emergency inpatient admissions came unaccompanied and 294 admissions came accompanied. In 14 admissions, the information on the accompaniment could not be safely extracted from documentation. 58.50% of the 123 unaccompanied patients were admitted involuntarily and out of all involuntarily admitted patients, 67.90% came unaccompanied. 51 (41.50%) emergency patients were not admitted involuntarily but came unaccompanied. This group of unaccompanied and voluntarily admitted patients had a mean age of 15.93 years (SD = 1.87, range 11–18 years), and 49.00% were male. Overall, 13.70% were refugees. The most common reasons for presentation were suicidal thoughts (64.70%), psychosocial burden (41.40%), externalized aggression (33.30%), depressive symptomatology (31.40%), deviant social behavior (27.50%), and non-suicidal self-injury (25.50%).

## Discussion

This study aimed to conduct an in-depth retrospective examination of children and adolescents admitted as emergency inpatients to a child and adolescent psychiatric hospital. In particular, we focused on the special subgroup of involuntarily admitted and (un)accompanied patients.

We analyzed the legal basis for emergency inpatient admissions and found that, out of 431 admissions, 24.6% of all emergency inpatient admissions were involuntary. In comparison, a Dutch registry study (2008–2017) showed that, out of 227 patients admitted on an emergency basis (aged 6–18 years), 137 (60.4%) were admitted voluntarily and 90 (39.6%) were admitted involuntarily [10]. A large retrospective collection of data from over 6 years in three German child and adolescent psychiatric hospitals found that 70.8% of patients were admitted voluntarily and 29.2% involuntarily [7]. However, this latter study evaluated all inpatient admissions, not only emergencies [7]. Differences between our data and the Dutch study may be explained by the different legal situations for involuntary admissions. In our study, of the 289 patients already known to the hospital, 71 (24.6%) were in involuntary inpatient emergency treatment. This shows that being a known patient at the hospital did not show a significant difference in whether an involuntary emergency inpatient admission occurred. Unfortunately, we are unable to make any statements about the extent to which these patients complied with the recommended regular treatment or whether they are only known to the hospital because of a previous emergency contact.

Over time, increase of bed capacities on the units and a higher rate of presented refugees were significantly associated with an increase in the number of inpatient admissions. For the number of involuntary inpatient admissions, time (in months) and the amount of refugees were significantly positively associated. In Germany, retrospectively collected rates of involuntary admissions of children (regardless of emergency status) decreased over the observation period from 32.4% (2004) to 25.7% (2008 and 2009) [7]. These results are not consistent with findings from Finland [21], where a noticeable increase in involuntarily admitted adolescents occurred [21]. It should be noted, however, that the study from Finland only examined 12–17 year-olds, whereas the German sample also included younger patients. In terms of average age, involuntarily admitted patients were significantly older than the voluntary patients in the above mentioned German cohort; three out of four involuntarily admitted adolescents were between 14 and 17 years old [7]. In addition, the overall frequency of admissions and rates of mandatory admissions vary widely across the EU for mentally ill patients [28]. In addition to legal bases, however, inpatient treatment capacity and other structures of psychiatric services must be taken into account [7, 28]. The number of child and adolescent mental health inpatient beds per 100,000 young people differs considerably between the European Union countries [29]. The number of beds varies from less than 2 per 100,000 young people in Sweden and Portugal to more than 50 beds per 100,000 young people in Germany and the Netherlands [29]. Further, available intensive outpatient child and adolescent psychiatric and psychotherapeutic care in the region may also be associated with fewer (involuntary) admissions. But also the children's and adolescents' attitudes and opinions towards the child and adolescent psychiatric hospitals could have an influence on the voluntary nature of treatment. If, for example, treatment in the hospital was presented as a threat or punishment before treatment, this could also explain patients' negative attitude [7]. Conversely, however, the helpful and empathic attitude of staff on duty in the emergency setting as well as child-friendly and welcoming equipment could also positively change this attitude. The combination of legal, political, economic, social, medical, methodological, and other factors involved in the involuntary placement or treatment of people with mental illness is complex and still poorly understood [28]. This aspect significantly complicates the comparability of findings from different countries.

We examined differences between voluntarily and involuntarily admitted patients. Involuntary inpatients were more likely to be male, though the proportion of girls still predominated; had a shorter length of stay; and had a higher frequency of refugees among them. In a previous German study with regular and emergency admissions, the two groups differed significantly in age and involuntarily admitted children were almost two years older [7]. The study also identified significant sex differences, with a higher percentage of males among voluntarily admitted children and adolescents (58.7%) compared to involuntarily admitted children and adolescents (53.8%) [7]. In contrast, a study conducted in the Netherlands reported a consistently high proportion of girls when comparing emergency patients admitted voluntarily (62.8%) to those admitted involuntarily (63.3%) [10]. A systematic review and meta-analysis found no association between sex and involuntary hospitalization despite high heterogeneity [30]. In a Finnish register study with adolescent boys, more involuntary psychiatric admissions were related to schizophrenia, substance use, and conduct disorders [31]. Among adolescent girls, these admissions were related to affective disorders and disorders related to somatic illnesses, including eating disorders [31]. In the present study, presenting due to alcohol consumption or deviant social behavior was significantly associated with involuntary admission. Therefore, although our results indicate that more female patients are admitted as voluntary and involuntary inpatients, similar to So et al. [10], the proportion of boys increased in the involuntarily treated group. This may result from more frequently reported aggressive or deviant social behavior as a reason for presentation in our study. A study in Finland retrospectively reviewed involuntary admissions of adolescents and found that involuntary admissions were associated with suicidal ideation [22]. In the present study, suicidal thoughts were associated with fewer involuntary admissions. As involuntary admissions had a higher amount of refugees and the majority out of these were male (94.6%), a greater number of boys, despite a higher overall percentage of female patients in involuntary admissions, is possible.

Unaccompanied refugee minors represent an at-risk group with a substantial prevalence of psychiatric morbidity [32, 33]. These young men have experienced numerous terrible events and likely lack social stability and support from their parents [32]. Particularly in emergencies, the language barrier is an additional complicating factor, making verbal de-escalation much more difficult. Involuntarily admitted minors in a German retrospective study received significantly shorter psychiatric inpatient treatment than voluntarily admitted patients [7]. In contrast to the aforementioned study (all admitted patients), we examined only patients admitted after an emergency, but showed the same trend. Shorter stays are common to cases that can be treated, stabilized, and de-escalated quickly (e.g., suicidal crises or substance intoxication) [7]. In addition, involuntarily admitted patients are often unwilling to undergo prolonged treatment.

A study from the Netherlands examining inpatient admission after emergency presentation reported that involuntary admission is associated with more serious psychiatric problems, higher risk of suicide, and earlier emergency involuntary admission [10]. In a large cohort study of children and adolescents in voluntary and involuntary psychiatric treatment (regular or emergency) in Germany, factors strongly associated with involuntary admission were being an adolescent (≥ 12 years old), substance abuse, psychotic disorders, intellectual disability, and hospitalization outside regular working hours [7]. Involuntary admissions to child and adolescent psychiatry (Finland) have also been reported to be related to outbursts of anger, damage to property, violent behavior, psychotic symptoms, and suicidal thoughts [22]. In a retrospective study in Italy on adolescents in psychiatric crises, involuntary treatment was particularly necessary for conduct disorders and comorbid diagnoses [23]. Consistent with our findings, these studies found that externalizing behaviors and substance abuse were associated with involuntary admission.

Data analyses have also shown that psychosocial burden is significantly associated with involuntary admission. However, Kaltialo-Heino did not identify an impact of familial adversities and sociodemographic factors in Finland, but the study examined all consecutive admissions regardless of emergency status [22]. This may explain differences, as we examined highly stressed patients who were admitted as inpatients directly after emergency presentation. If the family system is neither able nor willing to care for the patient (e.g., because of familial exhaustion), physicians may decide to admit the patient regardless of the clinical situation [10]. This is even more evident in our data, as 123 patients were admitted unaccompanied. The exact reasons for absence, perhaps also due to psychosocial burden, were not determined in this study but would be an important aspect for future research. However, patients presenting due to depressive symptomatology and school problems were less likely to be admitted involuntarily, whereas patients with externalizing behaviors had a higher likelihood.

The striking finding that 58.5% of the 123 unaccompanied patients were admitted involuntarily has led to our efforts to investigate the remaining group of 51 patients who came unaccompanied but were admitted voluntarily in more detail. This subgroup of patients consisted of older adolescents, and half of them were male. In general, lack of accompaniment by parents/custodians decreases the likelihood of sufficiently resolving a crisis outside regular working hours without an inpatient admission [11]. One in seven patients was a refugee, and the most common reasons for presentation were, in decreasing order, suicidal thoughts, psychosocial burden, aggression, depressive symptomatology, deviant social behavior, and non-suicidal self-injury.

Even though we obtained a large sample of more than 400 children and adolescents admitted on an emergency basis, some limitations should be considered. Due to retrospective data collection, documentation was not intended for research purposes, which may have resulted in the overestimation or underestimation of certain aspects. The physician collecting and scoring reasons were not blind to the type of admission, resulting in a potential for bias. Only the 4th quarter of each year was surveyed, which limits the generalizability to the whole year because seasonal fluctuations could not be taken into account. In particular, generalizability with regard to involuntary admissions has to be considered very carefully due to the different legal bases across countries.

The effort of staff to achieve voluntary acceptance of treatment and, if necessary, to carry out an involuntary placement often demands more time and personnel resources. Therefore, dealing with this clientele in an acute crisis is an important clinical challenge. These subgroups should be well characterized so that distinct treatment concepts can be tailored to them. Optimally, patients would no longer be in inpatient treatment against their will. All child and adolescent psychiatric hospitals should cope with these specific subgroups of emergency clients (involuntary inpatient admission, unaccompanied patients) and try to offer the best possible crisis intervention treatment despite the adverse circumstances. In the future, it would be important to determine to what extent children and adolescents benefit from treatment against their will and what the medium-term and long-term effects of such treatments are. If necessary, negative consequences should also be taken into account in future research.

## Data Availability

The raw data supporting the conclusions of this article will be made available by the authors without undue reservation.

## Declarations

### Human and animal participants

The studies involving human participants were reviewed and approved by the institutional examination board for the Medical Faculty of the University of Regensburg (Ethics Commission No. 19-1428-104).
